# TCR Fingerprinting and Off-Target Peptide Identification

**DOI:** 10.3389/fimmu.2019.02501

**Published:** 2019-10-22

**Authors:** Armen R. Karapetyan, Chawaree Chaipan, Katharina Winkelbach, Sandra Wimberger, Jun Seop Jeong, Bishnu Joshi, Robert B. Stein, Dennis Underwood, John C. Castle, Marc van Dijk, Volker Seibert

**Affiliations:** Agenus, Lexington, MA, United States

**Keywords:** T cell receptor, off-target prediction, cross-reactive peptides, MHC class I, NY-ESO^c259^

## Abstract

Adoptive T cell therapy using patient T cells redirected to recognize tumor-specific antigens by expressing genetically engineered high-affinity T-cell receptors (TCRs) has therapeutic potential for melanoma and other solid tumors. Clinical trials implementing genetically modified TCRs in melanoma patients have raised concerns regarding off-target toxicities resulting in lethal destruction of healthy tissue, highlighting the urgency of assessing which off-target peptides can be recognized by a TCR. As a model system we used the clinically efficacious NY-ESO-1-specific TCR C^259^, which recognizes the peptide epitope SLLMWITQC presented by HLA-A^*^02:01. We investigated which amino acids at each position enable a TCR interaction by sequentially replacing every amino acid position outside of anchor positions 2 and 9 with all 19 possible alternative amino acids, resulting in 134 peptides (133 altered peptides plus epitope peptide). Each peptide was individually evaluated using three different *in vitro* assays: binding of the NY-ESO^c259^ TCR to the peptide, peptide-dependent activation of TCR-expressing cells, and killing of peptide-presenting target cells. To represent the TCR recognition kernel, we defined Position Weight Matrices (PWMs) for each assay by assigning normalized measurements to each of the 20 amino acids in each position. To predict potential off-target peptides, we applied a novel algorithm projecting the PWM-defined kernel into the human proteome, scoring NY-ESO^c259^ TCR recognition of 336,921 predicted human HLA-A^*^02:01 binding 9-mer peptides. Of the 12 peptides with high predicted score, we confirmed 7 (including NY-ESO-1 antigen SLLMWITQC) strongly activate human primary NY-ESO^c259^-expressing T cells. These off-target peptides include peptides with up to 7 amino acid changes (of 9 possible), which could not be predicted using the recognition motif as determined by alanine scans. Thus, this replacement scan assay determines the “TCR fingerprint” and, when coupled with the algorithm applied to the database of human 9-mer peptides binding to HLA-A^*^02:01, enables the identification of potential off-target antigens and the tissues where they are expressed. This platform enables both screening of multiple TCRs to identify the best candidate for clinical development and identification of TCR-specific cross-reactive peptide recognition and constitutes an improved methodology for the identification of potential off-target peptides presented on MHC class I molecules.

## Introduction

T-cell receptors (TCRs) on T cells play a key role in adoptive T cell transfer, recognizing peptides displayed on major histocompatibility (MHC) complexes on the cell surface of, e.g., tumor-antigen presenting cells (APCs) (1). High TCR diversity ensures broad recognition and is a prerequisite for an effective immune system, as it is associated with efficient control of viral infections and other pathogens (2). Antigens are processed endogenously in the cell followed by the efficient binding of the peptide to MHC molecules and presentation of the antigen peptides on the cell surface. Foreign and neoantigen peptides presented on the cell surface on MHC molecules will be recognized by cytotoxic T-cells, specifically inducing cell death of infected cells or tumor cells. Recognition occurs through the heterodimeric cell-surface TCRs present on T cells, which feature highly variable alpha and beta chains. Somatic recombination processes occurring during thymus maturation result in high diversity of the alpha and beta TCR chains, giving rise to a large repertoire of unique TCRs providing a wide range of pathogen and tumor recognition (3). Through the process of TCR gene rearrangement, different checkpoints of negative and positive selection must be overcome to generate mature T cells with functional TCR diversity. Properly rearranged beta chains are first selected (beta selection), followed by the positive selection of TCRs capable of binding to MHC molecules and, finally, negative selection, in which TCRs with high affinity for binding self-peptides are depleted (4). Theoretical numbers for human TCR diversity range from 10^15^ to 10^20^ TCR clonotypes; however, the actual estimated TCR repertoire is <10^8^ in humans based on negative selection eliminating T cells recognizing self-peptides (5, 6). Therefore, T cell cross-reactivity is expected to cover an enormous number of pathogen peptides presented on the cell surface of APCs.

Identification of TCRs isolated and expanded from patient-derived tumor-infiltrating lymphocytes (TILs) (7, 8) or engineered TCRs with high affinity and specificity against tumor antigens have been used successfully in melanoma patients in clinical trials (9–11). Specific TCRs recognizing tumor-associated antigens isolated from patients with effective anti-tumor responses were transferred into T cells of melanoma patients, resulting in regression of metastatic tumor observed in 2/15 (13%) TCR-treated patients (9). Because of thymic selection, naturally occurring TCRs have low affinity to self-antigens, including self-antigens typically expressed on tumor cells, resulting in a low response rate of tumor patients (13%) treated with naturally occurring anti-tumor TCRs (12). Nevertheless, these successful results encouraged the identification and modification of novel TCRs recognizing various tumor-specific antigens (9, 10). However, using genetically modified high-affinity TCRs highlighted the risk of recognizing off-target peptide antigens presented on normal healthy tissues followed by major adverse events resulting in unintended tissue destruction triggering further secondary effects. Applying high-affinity MART-1 DMF5 TCRs in clinical trials against melanoma, 6/20 patients responded with off-tumor toxicities causing destruction of normal melanocytes in skin, uveitis and ototoxicity (10). High affinity-enhanced TCR a3a directed to MAGE A3 antigen on human leukocyte antigen HLA-A^*^01 initially revealed no off-target antigen effects tested in preclinical investigations; however, it resulted in lethal cardiac toxicity in patients treated with the affinity-enhanced MAGE A3 TCR a3a (13). Genetically engineered TCRs have demonstrated off-target toxicities leading to lethal effects in individual patients (14, 15), highlighting urgent need for improved methods to predict and identify potential epitopes recognized within healthy tissues in humans. Tools identifying cross-reactive self-antigens have been developed previously, e.g., “Expitope” (16), a tool based on RNA sequencing (RNA-seq) databases and “icrossr” (17) predicting all naturally possible self-antigens. However, these web tools do not consider the individual TCR recognition motifs and do not involve experimental *in vitro* data.

Here, we describe a method to predict potential cross-reactive peptides for genetically modified TCRs before entering a clinical trial. As proof of concept, we focused on a human-derived affinity enhanced NY-ESO^c259^ TCR recognizing the NY-ESO-1 and LAGE-1 derived peptide SLLMWITQC in complex with human leukocyte antigen serotype HLA-A^*^02:01 (18). NY-ESO^c259^ (GSK) is currently being evaluated in phase I/II clinical trials (NCT01567891) (19). NY-ESOc259-targeted T cell therapy has demonstrated efficacy in several forms of solid tumors and has not shown any indication for off-target toxicity to date, making it a good subject to understand the relationship between off-target peptide prediction and the translation into real off-target toxicity. Both NY-ESO-1 antigen and NY-ESO-1 specific T cells have been extensively studied and found to generate a T cell response and anti-tumor effects, respectively (11, 20). To investigate our methods for off-target peptide prediction, we used a modified version of the wild-type peptide (SLLMWITQC) with a cysteine (C) to valine (V) substitution at position nine (P9). It has been shown previously that this modification enhances the ability of the epitope to be recognized *in vitro* by the TCR, through tightening the binding of the modified peptide to HLA-A^*^02 and increasing TCR affinity (21, 22). These two properties lead to a stronger T cell-mediated cytolysis and activation without compromising cross-reactivity with the wild-type peptide (22). Using this antigenic peptide (SLLMWITQV) as a starting point, we substituted every single position except the anchor positions P2 and P9 within the 9-mer epitope peptide with all 19 possible alternative amino acids to generate a library of 134 peptides (133 altered peptides plus epitope peptide). Each individual peptide was evaluated using functional assays analyzing binding of the TCR to the peptide-MHC complex, activation of the T cells after recognition of peptide-MHC (pMHC) complex and killing of the APCs. We constructed Position Weight Matrices (PWMs) for the three *in vitro* assays by assigning normalized experimental measurements to each of the 20 amino acids in each position. The generated PWMs were applied to 336,921 HLA-A^*^02:01 binding 9-mer peptides (predicted by netMHCpan v3.0 and defined based on IC50 score ≤ 500 nM) to predict peptides with high TCR recognition score. Experimental validation of high scoring peptides identified 7 antigens (including the cognate NY-ESO-1 antigen SLLMWITQC) and demonstrated strong activation of primary T cells expressing the NY-ESO^c259^ TCR. The proposed method is a first step toward validation of TCR safety.

## Materials and Methods

### Cell Lines and Plasmids

Tap-deficient T2 (174x CEM.T2) cells and HEK293T cells were purchased from ATCC and maintained in RPMI 1640 (BioConcept) supplemented with 10% fetal calf serum (FCS; HyClone), 2 mM L-glutamine and 1% Penicillin/Streptomycin at 37°C and 5% CO_2_. The EGFP reporter cell line was based on a murine TCR negative thymoma cell line derived from strain BW5417 (ATCC^®^TIB-47TM) and was stably transduced with (i) TCR NY-ESO^c259^ (International Patent Application Publication No. WO2017044672A1) in which the human constant regions were replaced with those of mouse, (ii) a chimeric mouse/human CD8 as well as (iii) an EGFP reporter construct linked to a minimal IL-2 promoter comprising three NFAT-binding sites (3xNFAT) (23). The transduced cells were termed NY-ESO^c259^ AKD10R3 cells and will be called effector cells below. Mouse cell lines were cultured in SF-IMDM (BioConcept) supplemented with 3% FCS, 1% Penicillin/Streptomycin, and 50 μM beta-mercaptoethanol at 37°C and 10% CO_2_.

### Peptides

Peptides (Peptides & Elephants, or synthesized in-house) were dissolved in dimethyl sulfoxide (DMSO) at concentrations of 2–10 mg/mL, aliquoted and stored at −80°C. Peptide-pulsing experiments were performed by pre-incubating T2 cells with 25 μg peptide per 1 × 10^6^ cells in PBS for 2 h at 37°C and 5% CO_2_, washed three times to remove unbound peptides and used for further downstream analysis.

### Immunoassays Measuring Binding, Activation, and Killing

#### Binding

Peptide-pulsed T2 cells were simultaneously stained with anti-HLA-A2 clone BB7.2-APC (eBiosciences, 1:500 dilution) and soluble NY-ESO^c259^ TCR-PE (in-house production) for 30 min at room temperature (RT). Cells were washed twice with washing buffer before FACS-analysis using a BD FACS Calibur. Data analysis was performed using FlowJo V10 software.

#### Killing Assay

Peptide-pulsed T2 cells were incubated with effector cells in a 1:5 ratio overnight in SF-IMDM at 37°C and 10% CO_2_. For cell-surface staining, cells were washed twice and incubated with anti-human CD4-PE (BD Pharmingen, 1:100 dilution) for 30 min at RT, washed twice and fixed for intracellular staining using the Fixation/Permeabilization Solution Kit (BD Cytofix/Cytoperm™) according to the manufacturer's instructions. Cells were washed twice with 1x BD Perm/Wash™ Buffer and intracellular staining was performed by incubating the cells with anti-active caspase-3 clone C92-605-Alexa Fluor^→^ 647 (BD Pharmingen, 1:200) for 40 min at 4°C. Finally, cells were washed twice with 1x BD Perm/Wash™ buffer before resuspending in washing buffer for FACS-analysis (BD FACS Canto II). Data analysis was performed using FlowJo V10 software.

#### Activation Assay

Peptide-pulsed T2 cells were incubated with effector cells in a 2:1 ratio overnight in SF-IMDM at 37°C and 10% CO_2_, washed twice and stained with anti-mouse TCR-^®^ chain clone H57-597 (BD Pharmingen, 1:500) for 30 min at RT. Cells were washed twice before FACS-analysis using a BD FACS Canto II. Data analysis was done using FlowJo V10 Software.

### Generation of Soluble TCR

Expression constructs for soluble TCR alpha and beta chains were synthesized (Genscript and ThermoFisher Scientific) and cloned in pCDFDuet-1 and pETDuet-1, respectively. Jun-Fos domains were introduced to facilitate the heterodimerization of alpha and beta chains (24, 25). Soluble NY-ESO^c259^ TCR was prepared as described in Boulter et al. (25). For cell staining, soluble TCR was tetramerized with streptavidin-PE conjugate.

### PCR Amplification and *in vitro* Transcription

PCR amplification from the TCR NY-ESO^259^ construct containing the T7 promoter was performed applying the Phusion^®^ High-Fidelity DNA Polymerase (NewEngland BioLabs) using the forward primer 5′-GTC GAC TAA TAC GAC TCA CTA TAG GGA GAA AGC-3′ and the reverse primer 5′-GCA ATG AAA ATA AAT GTT TTT TAT TAG GCA GAA TCC-3′ (Microsynth). Initial denaturation step was performed at 98°C for 1 min followed by 40 PCR cycles, consisting of a denaturation step at 98°C for 10 s, an annealing step at 65°C for 3 s, an extension step at 72°C for 10 s and a final extension step at 72°C for 5 min. PCR reactions were purified using the NucleoSpinGel and PCR Cleanup Kit from Macherey-Nagel. *In vitro* transcription was performed using HiScribe™ T7 ARCA mRNA Kit (with tailing; NEB) following the manufacturer's protocol for mRNA Synthesis with Modified Nucleotides. RNA was purified with the RNeasy MinElute Cleanup Kit (Qiagen) and quality of the RNA was confirmed using a Bioanalyzer 2100 (Agilent RNA 6000 Pico).

### Peripheral Blood Mononuclear Cells (PBMCs) Isolation, Activation, and Electroporation

PBMCs obtained from healthy donors were isolated from a buffy coat obtained from “Blutspendezentrum SRK beider Basel” using Ficoll-Paque plus centrifugation (GE Healthcare) and cultured in T cell media (RPMI + 10% huFCS, 0.8 mM L-Glutamine, 1x MEM non-essential Amino Acids, 1x MEM Amino Acid Solution, 10 mM HEPES, beta-2-Mercaptoethanol, 50 IU/ml human IL-2, 2.5 ng/ml human IL-7). To activate cells, a cocktail of human CD3/CD28/CD2 T Cell Activator (ImmunoCult) was added. 1 × 10^7^ cells were electroporated with 10 μg of TCR NY-ESO^c259^ RNA following the Neon transfection system guide. Cells were resuspended in pre-warmed T cell media directly after electroporation to a final concentration of 2.5 × 10^7^ cells/ml and incubated at 37°C and 5% CO_2_. After 2 h cultivation, 0.1 μg/ml DNase I was added. TCR expression was analyzed 24 h post-electroporation by staining with iTAG NY-ESO Tetramer/APC HLA-A^*^02:01 (SLLMWITQC; MBL).

### Primary T Cell Activation Assay

Peptide pulsed T2 cells were incubated with effector T cells in a 2:1 ratio overnight in T cell medium at 37°C and 5% CO_2_. Cells were washed twice and stained with anti-human CD25-APC antibody (BD Biosciences; 1:20 dilution) and Human Fc Block (eBioscience, 1:100 dilution) for 30 min at 4°C and analyzed using a BD FACS Canto II. Data analysis was done using FlowJo V10 Software.

### Statistical Analysis of Experimental Data and Predictive Model Building

A statistical analysis and position weight matrix (PWM) building was performed independently for each of the tested assays: binding, activation and killing.

A PWM was constructed by assigning a normalized experimental value (*W*_*a, i*_) to each of the 20 amino acids in each position. The value *W*_*a, i*_ for each amino acid *a* at each position *i* was calculated as follows:

(1) $$\begin{array}{l} W_{a,i}=\frac{L_{a,i\;}}{L_{epitope}}*100\\ \;a\in \left[A,R,N,D,C,Q,E,G,H,I,L,K,M,F,P,S,T,W,Y,V\right],\\ \;i\in \left[1,3,4,5,6,7,8\right] \end{array}$$

where *L* indicates the mean of replicated experimental measurements, *L*_*epitope*_ denotes the measurement of the epitope peptide. For each replicate, we subtracted the corresponding background noise value prior to calculating the mean and normalizing to the epitope peptide signal. Calculated *W*_*a, i*_ values exceeding 100% were trimmed to 100% and negative *W*_*a, i*_ values were adjusted to zero.

The quantitative PWM score for a query sequence is the sum of the PWM values for each amino acid in the sequence, normalized to the score of the epitope peptide:

(2) $$\begin{array}{l} S_{seq}=\;\frac{\sum_{i\in P}W_{l_{i},i}}{S_{epitope}},\;P=\left\{1,3,4,5,6,7,8\right\} \end{array}$$

here *S*_*seq*_ is a score of the query sequence, *S*_*epitope*_ denotes the score of the epitope peptide sequences which is equal to the maximum possible score for PWM, and *l*_*i*_ stands for the amino acid at position *i* in an input sequence. Anchor positions two and nine were excluded from the calculation of the score.

All data analyses were done using custom scripts implemented in the R (version 3.4.0) programming language (26). The majority of figures were generated using the ggplot2 package (27). ROC curve analysis was performed using the ROCR package (28).

To use alanine scan data, where each peptide position is tested with only an alanine substitution rather than all amino acids, we adapted the algorithm whereas a successful alanine substitution marked positions putatively allowing any amino acid. The algorithm propagated the resulting position-specific motif as described above, scoring peptides from the human proteome to identify hits.

### HLA Binding Affinity Prediction

The binding affinity of 9-mer peptide sequences to HLA-A^*^02:01 was calculated using the command-line version of the netMHCpan 3.0 software tool (29). It was used as a part of the IEDB MHC-1 binding prediction tools set (30). The human protein sequences in FASTA format were retrieved from the NCBI Reference Sequences Database (RefSeq) (31) and used as an input for netMHCpan 3.0.

### Gene Expression Data

RNA expression levels across 37 normal human tissues based on RNA sequencing data were downloaded from The Human Protein Atlas (http://www.proteinatlas.org/about/download) (32).

## Results

### Experimental Validation of TCR NY-ESO^c259^ Specificity

Adoptive T cell therapy with genetically modified TCRs has shown encouraging results treating patients with tumors (11). However, generation of higher-affinity TCRs and therapeutic use of engineered TCRs has triggered adverse side effects such as autoimmune toxicity, leading to destruction of healthy tissues and lethal effects on treated patients (13). Earlier efforts to identify off-target peptides to validate genetically modified TCRs are based on *in vitro* cell based studies using cell lines endogenously expressing the antigen and *in vivo* studies using mouse models.

**Figure 1:**
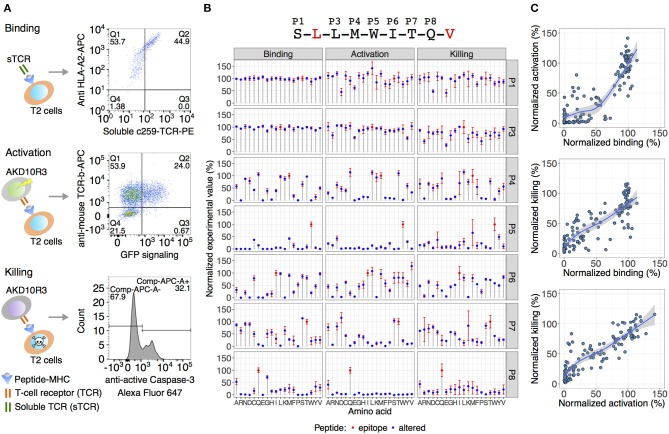
Experimental validation of TCR NY-ESO^c259^ specificity. **(A)** An overview of the assays used for TCR specificity assessment analyzing binding of the TCR to the peptide-MHC complex on antigen-presenting T2 cells, activation of TCR-expressing cells by co-culturing with T2 cells and final killing of the target T2 cells. **(B)** The effect of amino acid substitution in the epitope peptide (displayed at the top of the figure) on TCR binding/activation/killing. Blue dots depict the average signal of at least two independent experimental replicates (binding *n* = 3, activation *n* = 3, killing *n* = 2). The error bars show standard error of the mean (SEM). An observed TCR NY-ESO^c259^ specificity pattern is similar across all three assays. Positions five (P5) and eight (P8) are crucial for TCR sensing of the peptide, and to a lesser extent, positions P4, P6, and P7. **(C)** Pairwise comparisons of the normalized experimental values among the three assays. Activation and killing assays yielded a strong linear relationship (*R*^2^ = 0.8, *p* < 2e-16) indicating a strong dependence between T cell activation level and killing efficiency. Comparing binding with activation and killing assays resulted in a less linear relationship, and in many cases at least 75% binding efficiency is required to trigger T cell activation and killing mechanisms.

Here, we focused on developing a novel approach to identify off-target peptides exemplified by an affinity enhanced TCR NY-ESO^c259^ targeting NY-ESO-1/LAGE-1-derived SLLMWITQC peptide in complex with HLA-A^*^02:01. TCR NY-ESO^c259^ is in phase II clinical trial for use in patients with non-small-cell lung cancer (NCT03709706). Antigenic peptides usually vary in length from 8 to 10 amino acids. In case of class I MHC HLA-A^*^02:01 specific peptides, nine amino acid peptides are preferably presented on the surface and the specificity of peptide binding is based on two “anchor” positions defined as leucine (L) at position two (P2) and valine (V) or leucine (L) at position nine (P9) (33–35). To improve binding of the peptide to MHC complex and therefore stimulation of tumor-reactive T cells, we used peptides accommodating a valine (V) at position 9 instead of a cysteine (C) (21). Using altered peptides replacing every position in the 9-mer peptide, except the anchor positions P2 and P9, with all 19 possible alternative amino acids resulted in a total set of 134 peptides including the SLLMWITQV epitope peptide. We left anchor residues constant to focus on substitutions more likely to directly interact with the TCR, rather than substitutions more likely to interact with the presenting HLA. We developed three independent cell-based *in vitro* assays to interrogate the contribution of each single amino acid at each position, enabling us to apply the TCR NY-ESO^c259^ recognition kernel (the TCR fingerprint) to the human proteome. This includes a binding assay to determine the binding efficiency of the peptides to TCR NY-ESO^c259^, an activation assay to measure activation of the TCR-expressing cells, and a killing assay of the target cells by flow cytometry (Figure 1A).

TAP-deficient T2 (174x CEM.T2) cells lack the TAP-dependent transport of cytosolic derived peptides to enter the endoplasmic reticulum, resulting in accumulation of empty MHC class I molecules on the cell surface (36). Thus, exogenous peptides can be loaded and presented on T2 cells to form a peptide-MHC I complex. Accordingly, T2 cells were pulsed with each of the 133 altered peptides as well as the SLLMWITQV epitope peptide, and binding to TCR NY-ESO^c259^ was measured by simultaneously staining the peptide-presenting T2 cells with soluble fluorescently labeled TCR NY-ESO^c259^ and anti-HLA-A2 (Figure 1A).

To measure activation of TCR NY-ESO^c259^ expressing cells, we stably introduced a chimeric version of the TCR NY-ESO^c259^, a chimeric mouse/human CD8, as well as an EGFP reporter construct linked to a minimal IL-2 promoter comprising three NFAT binding sites (3xNFAT) to a mouse TCR-negative thymoma cell line derived from strain BW5417 (ATCC^®^TIB-47TM) (23). The transduced cells were termed NY-ESO^c259^ AKD10R3 cells. T cell activation triggers signaling pathways inducing the transcription of genes from the IL-2 promoter. EGFP expression reflects the TCR-dependent activation of the NY-ESO^c259^ AKD10R3 cells measured by flow cytometry. Peptide-pulsed T2 cells were co-cultured with NY-ESO^c259^ AKD10R3 cells and TCR-dependent T cell activation was measured by quantifying the percentage of EGFP-expressing cells (Figure 1A). Applying a similar co-culture-setup, we employed target cell killing by measuring caspase3/Fas-mediated cell killing and simultaneously gating for CD4-positive target T2 cells using FACS-analysis (Figure 1A). We normalized the resulting percentages of positive cells from each of the three experimental assays (binding, activation and killing) to the epitope peptide's signal and generated a TCR-recognition kernel defining the bias toward specific amino acid residues to provide a comprehensive TCR-recognition motif assessment (Figure 1B).

Based on the *in vitro* analyses given the percentage of positive cells for each of the three methods (binding, activation, and killing), positions P1 and P3 were determined to be insensitive to variation, positions P5 and P8 were found to be crucial, and to a lesser extent, positions P4, P6, and P7 played a role in recognition of the NY-ESO^c259^ TCR (Figure 1B). Pairwise comparison of the activation and killing assays yielded a strong linear relationship between two assays (*R*^2^ = 0.8, *p* < 2e-16), indicating a strong dependency between T cell activation and killing efficiency (Figure 1C, lower panel). Comparing binding with activation and killing assays resulted in a less linear relationship and in many cases at least 75% binding efficiency is required to trigger T cell activation and killing mechanisms. Remarkably, high binding efficiency of the peptide to the soluble TCR NY-ESO^c259^ does not necessary result in high activation or killing activities, exemplified by peptides with 100% binding efficiency and substantially lower activation and killing efficiencies (~50%) (Figure 1C, upper and middle panel). In addition, the opposite effect was observed for binding: some peptides with low binding efficiency (<50%) yielded high activation and killing efficiency (>50%) (Figure 1C, upper and middle panel).

### Position-Specific TCR Specificity

**Figure 2:**
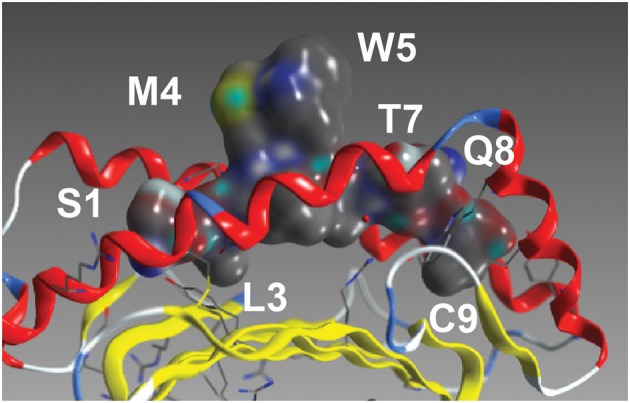
The NY-ESO peptide. The NY-ESO-1/LAGE-1-derived SLLMWITQC peptide in complex with HLA-A^*^02:01 (22). Visible amino acids and their peptide position are labeled.

**Figure 3:**
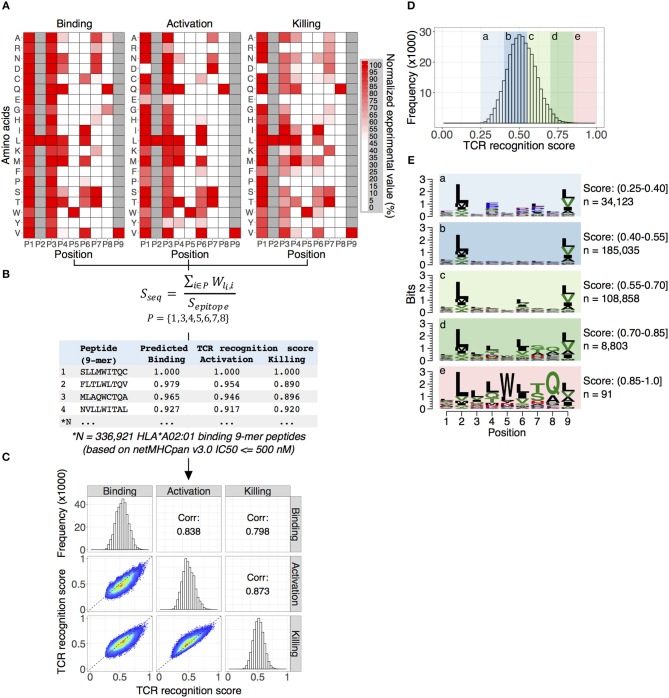
Predicting TCR NY-ESO^c259^ cross-reactive antigens. **(A)** Position weight matrices (PWMs) are constructed by assigning a normalized experimental value to each of the 20 amino acids (rows) at each position (columns). The generated PWMs represent the TCR NY-ESO^c259^ fingerprint profile: a unique pattern for TCR recognition of the peptide-MHC complex. A high normalized value corresponds to amino acid substitution having little effect on TCR recognition of the peptide-MHC complex. Tryptophan (W) at position five (P5) and glutamine (Q) at position eight (P8) are crucial for TCR NY-ESO^c259^ recognition of the peptide-MHC complex and are part of the TCR recognition motif. In general, we observed a high similarity between PWMs for three assays (binding, activation, and killing), though some prominent differences for specific amino acids for specific positions exist, e.g., replacement of serine (S) at P1 with aspartic (D) or glutamic (E) acids has no effect on TCR binding to peptide-MHC complex but has a substantial effect on activation and killing. **(B)** Scoring 336,921 HLA^*^A02:01 binding 9-mer peptides with PWMs. Binding affinity of individual peptides to HLA^*^A02:01 determined by software package netMHCpan v3.0 and defined as IC50 score ≤ 500 nM. Anchor positions (P2 and P9) were excluded from the calculation of the TCR recognition score. **(C)** The distribution of the TCR recognition scores for individual assays. Predicted scores follow a Gaussian distribution with the peak around score 0.5 (diagonal panel). A pairwise comparison (lower left panel) of the predicted scores demonstrates a high Pearson correlation coefficient (upper right panel) between all tested assays. **(D)** Calculating overall TCR recognition score by taking the mean of the predicted scores estimated for binding, activation and killing assays. All peptides were separated into five groups ranked by overall TCR recognition score. **(E)** The peptides with low recognition score demonstrate no evidence of specific amino acid enrichment in any position, except anchor position (P2 and P9) enriched for leucine (L) and valine (V) as expected for HLA-A^*^02:01 binding peptides (panels a–d). The group of peptides with highest predicted score displayed a strong enrichment of TCR NY-ESO^c259^ recognition motif, specifically an enrichment of tryptophan (W) at position five (P5) and glutamine (Q) at position eight (P8), which fits the observed experimental data (panel e).

As with most HLA-A^*^02 bound peptides, the two anchor residues at P2 and P9 face into the MHC molecule toward the beta-sheet floor, wedged between the alpha 1 and 2 helices (Figure 2) (22). The residues P3 and P6, adjacent to P4 and P5, respectively, likely interact with each other and the MHC, orienting the TCR engaging residues P4 and P5 (M,W) residues away from the pMHC complex toward the TCR. In addition to this MW peg, the P8 (Q) residue is TCR-facing. Going beyond alanine scans, the results in Figure 3A enable interrogation of the impact of all amino acids at each position on TCR recognition.

Alanine scans substitute only alanine at each position. Examining published alanine scans from the TCR recognizing the MAGE-A3 HLA A^*^01:01 epitope EVDPIGHLY (13), the top three hits are peptides EVDPIGHVY (MAGE-A6), EVDPIRHYY (MAGE-B18) and ESDPIVAQY (TTN), where the latter is the peptide associated with the off-target toxicity (13). Additionally, we mimicked an alanine scan using the alanine substitution activation values for our c259 full substitution screen. Using the full substitution matrix, the peptides FLTLWLTQV and QVFLWLAQV, ranking first and fourth, show activation almost 150 and 130% that of the NYESO1 peptide. Using the alanine scan only, the peptides ranked 200 and 261, respectively. These peptides have five and seven edits from the NYESO1 peptide, respectively. Thus, while alanine scans can identify off-target peptide activation, leveraging the full substitution matrix successfully ranks peptides with high activation.

P1 (S) and P3 (L) are relatively insensitive to variations. However, P1 (S) acidic replacements (E and D) decrease activation and killing. Replacement of a neutral but hydrophilic side chain at P1 (S) with negatively charged acidic residues may cause perturbations in the formation of the ternary complex. P4 (M) shows less tolerance for substitutions, with most replacements decreasing binding, activation and killing. P5 (W) is a key interaction with the TCR (Figure 2). P5 (W) is not even tolerant of substitution by other aromatics F and Y known to be functional replacements from protein folding and mutation studies. P6 (I) faces the MHC and, while likely not directly involved in TCR binding, it is likely involved in pMHC conformational structure. Conservative changes L and V maintain binding, activation and killing. Surprisingly, C, M, S, and T substitutions also provide robust TCR activation. P7 (T) is particularly tolerant to S, K, D substitutions. P8(Q) faces the TCR (Figure 2) and shows little tolerance to variation.

### Predicting TCR NY-ESO^c259^ Cross-Reactive Antigens

We next developed a computational approach that uses *in vitro*-generated information from the screening of the library of 134 peptides (133 altered peptides plus epitope peptide) to rank known 9-mer peptides predicted to bind HLA-A^*^02:01 according to the predicted NY-ESO^c259^ TCR recognition potential. Our strategy is outlined in Figure 3.

First, a Position Weight Matrix (PWM) is constructed independently for each assay by assigning a normalized experimental value to each of the 20 amino acids (rows) at each position (columns). The generated PWMs represent a TCR NY-ESO^c259^ fingerprint profile—a unique pattern for TCR recognition of the peptide-MHC complex (Figure 3A). In general, we observe a high degree of similarity between TCR NY-ESO^c259^ fingerprint profiles obtained for individual experimental assays. We quantified the similarity between PWMs from three assays (binding, activation, and killing) using Pearson correlation coefficient optimized for pairwise comparison of PWMs (37). High correlation coefficient scores of 0.80, 0.78, and 0.84 were obtained for binding vs. activation, binding vs. killing, and activation vs. killing assays, respectively. Nevertheless, despite significant similarity between three assays, prominent differences for some positions for specific amino acid exist. For example, a substitution of serine (S) in the position P1 by any other amino acid does not affect binding of TCR NY-ESO^c259^ to the peptide-MHC complex, which is evident from the binding assay; however, we observe a significant decrease in T-cell activation and killing efficiency when serine (S) is replaced with aspartic (D) or glutamic (E) acids (Figure 3A). Similarly, a substitution of leucine (L) at position P3 to either arginine (R), glutamic acid (E), lysine (K), or proline (P) leads to a substantial decrease in killing efficiency but has a less prominent effect on T cell activation and no effect on binding efficiency (Figure 3A).

Second, the PWMs were used for scoring HLA-A^*^02:01 binding 9-mer peptides (Figure 3B). We calculated a binding affinity (IC50, nM) of all human 9-mer peptide sequences (10,470,365) to the HLA-A^*^02:01 molecule using netMHCpan v3.0—an artificial neural networks (ANNs) based software package trained on more than 180,000 quantitative binding data (38). Subsequently, we discarded peptides with low binding affinity (IC50 > 500 nM) and obtained a final list of 336,921 9-mer peptide sequences with intermediate and high binding affinity toward HLA-A^*^02:01. While we expected to identify candidate peptides with many amino acid edits from the original target peptide, we started by summing the independent first order changes; therefore, the peptide score is the sum of position-specific scores for each of the amino acid composing the peptide (Figure 3B) (39). Additionally, we normalized the score of every peptide to the score of the epitope sequence. Predicted scores follow a Gaussian distribution with the peak around score 0.5 (Figure 3C, diagonal panel). We observed a high pairwise correlation between the predicted scores from the three assays, with Pearson correlation coefficients of 0.84, 0.80, and 0.87 for binding vs. activation, binding vs. killing, and activation vs. killing, respectively (Figure 3C, upper and lower panels).

Third, given the high similarity of the predicted scores obtained from three different methods, we calculated a NY-ESO^c259^ TCR recognition score for each peptide by taking the mean of the predicted scores estimated for binding, activation, and killing assays (Figure 3D). Therefore, the TCR recognition score aggregates the predictive power of the three tested assays, providing a single measure for a peptide's cross-reactive potential. It was evident from the experimental data (Figures 1B, 3A), that tryptophan (W) at position P5 and glutamine (Q) at position P8 are crucial for TCR NY-ESO^c259^ recognition of peptide-MHC complex and are part of the TCR recognition kernel. Also, an alanine (A) at position P8 could partially preserve TCR recognition of peptide-MHC complex, but at a significantly lower level in comparison with glutamine (Figure 1B). We sought to find if our prediction algorithm correctly assigns a high predicted score to the peptides enriched for TCR recognition kernel. To verify this, we separated peptides into five groups ranked by NY-ESO^c259^ TCR recognition score and generated a sequence motif for each group (Figure 3E). The low scoring peptides (score 0.25–0.4) showed no evidence of specific amino acid enrichment in any position, except anchor positions (P2 and P9) enriched for leucine (L) and valine (V) as expected for HLA-A^*^02:01 binding peptides (Figure 3E, panels a–d) (33, 34). In contrast, the group of highest scoring peptides (score 0.85–1) displayed strong enrichment for the TCR NY-ESO^c259^ recognition motif, specifically an enrichment of tryptophan (W) at position P5 and glutamine (Q) at position P8, which fits the observed experimental data (Figure 3E, panel e). We also observed an increase of alanine at the position P8, as this amino acid can be partially tolerated by the TCR NY-ESO^c259^.

### Validation of NY-ESO^c259^ TCR Predicted Peptides in Primary T Cells and AKD10R3 Cells

**Figure 4:**
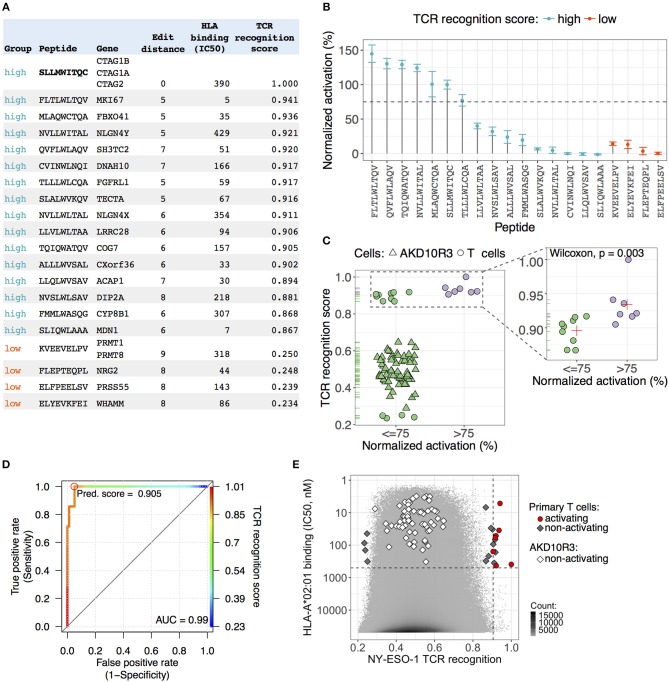
Validation of NY-ESO^c259^ TCR predicted peptides in primary T cells and AKD10R3 cells. **(A)** An overview of the peptides selected for validation in primary T cells. An edit (Hamming) distance, the minimum number of operations required to transform a peptide sequence into an epitope peptide sequence (SLLMWITQC), is depicted. HLA binding affinity (IC50, nM) was predicted by the netMHCpan v3.0 software package. The TCR recognition score integrates the predictive power of the three assays, calculated by taking the mean of the predicted scores from binding, activation and killing assays, providing a single measure for the peptide cross-reactive potential. **(B)** Experimental validation of the predicted peptides in primary T cell activation assay. Peptide pulsed T2 cells were co-cultured with effector T cells expressing TCR NY-ESO^c259^ and T cell activation was measured by quantifying the percentage of CD25 positive cells. Background noise was subtracted from activation measurements and individual values were normalized to the epitope peptide signal. The dots depict the average signal of three independent experimental replicates. The error bars show standard error of the mean. Seven peptides (including epitope peptide SLLMWITQC) from the group of peptides with high predicted score demonstrated at least 75% activation of primary T cells. All peptides in the group with low predicted score demonstrated either weak (<15%) activation or no activation in T cells. **(C)** All tested peptides: (1) 65 peptides with intermediate TCR recognition scores tested without replicates in AKD10R3 cells and (2) 20 peptides with high and low recognition scores tested in primary T cells, were separated in two groups based on 75% activating threshold (left panel). Among the peptides with high predicted score (right panel), activating peptides (>75%) on average had a higher predicted score (0.923) than non-activating (0.896) peptides and the observed differences were statistically significant (Wilcoxon two-sided test, *p* = 0.003). The red cross depicts the mean signal of the group. **(D)** Receiver operating characteristic (ROC) curve showing the performance of linear classifier to discriminate between activating (>75% normalized activation) and non-activating (≤ 75% normalized activation) peptides based on TCR recognition score parameter. A high value (0.99) of the area under the ROC curve (AUC) indicates a good performance of the parameter to distinguish between two groups of peptides. The color key visualizes the mapping from TCR recognition score cut-offs. The optimal cut-off score value, maximizing the sensitivity of the classifier is marked by red circle. **(E)** The distribution of TCR recognition scores (x-axis) and HLA-affinity binding values (IC50, nM) (y-axis) for all human 9-mer peptides (10,470,365). Peptides tested in activation assay in primary T cells and AKD10R3 cells depicted as geometric figures. All peptides with intermediate and low recognition scores showed either no evidence of cell activation or very weak activation. Seven peptides (including NY-ESO-1 antigen SLLMWITQC) with high predicted score demonstrated strong activation of primary T cells. A vertical dashed line is an estimated cutoff of 0.905 for TCR recognition score separating activating peptides from non-activating peptides. A horizontal dashed line depicts the IC50 value of 500 nM separating peptides with high binding affinity to HLA^*^A02:01 from peptides with low binding affinity.

We sought to find a cutoff value for the predicted NY-ESO^c259^ TCR recognition score separating cross-reactive antigens from the rest of the peptides without cross-reactive potential. To determine a cutoff value, we sampled peptides with different recognition scores from the whole distribution of the scores (Figure 3D) and tested them in activation assays in human primary T cells and AKD10R3 cells. First, we selected 16 peptides (including NY-ESO-1 antigen SLLMWITQC) with the highest scores and 4 peptides with the lowest scores and assessed their activating potential in primary T cells (Figure 4A). Peptide-pulsed T2 cells were co-cultured with effector human primary T cells containing introduced TCR NY-ESO^c259^, and T cell activation was measured by quantifying the percentage of CD25 positive cells. We subtracted background noise from experimental measurements and normalized individual values to the epitope peptide signal (SLLMWITQC). Out of 16 peptides with high recognition scores, 44% (7/16) demonstrated at least 75% activation (6, including the original peptide, demonstrated activation above 100%), 27% (4/16) were demonstrated activation in the range of 20–40%, and 33% (5/16) displayed either weak (<15%) activation or no activation at all (Figure 4B). All the peptides in the group with low recognition score demonstrated either very weak (<15%) activation or no activation at all (Figure 4B). Second, we tested a larger pool of 65 peptides sampled from the middle part (ranging from 0.3 to 0.65) of the NY-ESO^c259^ TCR recognition score distribution for activating AKD10R3 cells (Supplementary Table 1). The binding of these peptides to the HLA-A^*^02:01 molecule was previously validated by nano-capillary electrospray ionization-tandem mass spectrometry (40). The activation assay was performed as previously described for the library of 133 altered peptides plus epitope peptide, and individual experimental measurements were normalized to the epitope (SLLMWITQC) peptides signal after the background correction. None of the tested peptides showed evidence of meaningful AKD10R3 cell activation, with a median activation of 1.2% across 65 tested peptides.

We split all tested peptides into two groups (activating and non-activating) based on a 75% activation threshold and checked the difference in the predicted NY-ESO^c259^ TCR recognition score between groups (Figure 4C, left panel). We observed a clear separation between activating (>75%) and non-activating (≤ 75%) peptides in the TCR recognition score (Figure 4C, left panel). Next, we focused on the peptides with high score as we aimed to find a cutoff separating cross-reactive antigens from the rest of the peptides. The activating peptides on average had a higher score (0.923) than non-activating (0.896) peptides and the observed difference was statistically significant (Wilcoxon two-sided test, *p* = 0.003) (Figure 4C, right panel). We built a linear classifier discriminating between activating and non-activating peptides based on the predicted NY-ESO^c259^ TCR recognition score parameter with the very high accuracy (0.953) (Figure 4D). We selected a cutoff of 0.905 maximizing the sensitivity (1) of the classifier, thereby minimizing the number of false negatives (Figure 4D). The specificity of the classifier at the selected cutoff was also very high (0.950).

We were interested to find how many 9-mer peptides from the whole human proteome might constitute potential cross-reactive antigens for NY-ESO^c259^ TCR. We detected 12 peptides with predicted scores greater than the cutoff (0.905) with predicted HLA binding affinity (IC50) ≤ 500 nM (Figure 4E). We had previously tested 11 of these peptides in the activation assay in primary T cells and 7 of them (including NY-ESO-1 antigen SLLMWITQC) demonstrated strong activation of T cells (Figures 4B,E). Notably, these peptides demonstrate an unexpectedly high degree of sequence dissimilarity to the epitope peptide sequence (SLLMWITQC). We calculated the edit (Hamming) distance, the minimum number of substitutions required to transform a peptide sequence into an epitope peptide sequence and found that the majority of peptides (6/7) had an edit distance of at least five, and one peptide had an edit distance of seven. This finding indicates that peptides with sequences very different from the epitope peptide sequence can still be recognized by NY-ESO^c259^ TCR and trigger activation of T cells.

### Estimating Biological Relevance of NY-ESO^c259^ TCR Activating Peptides

**Figure 5:**
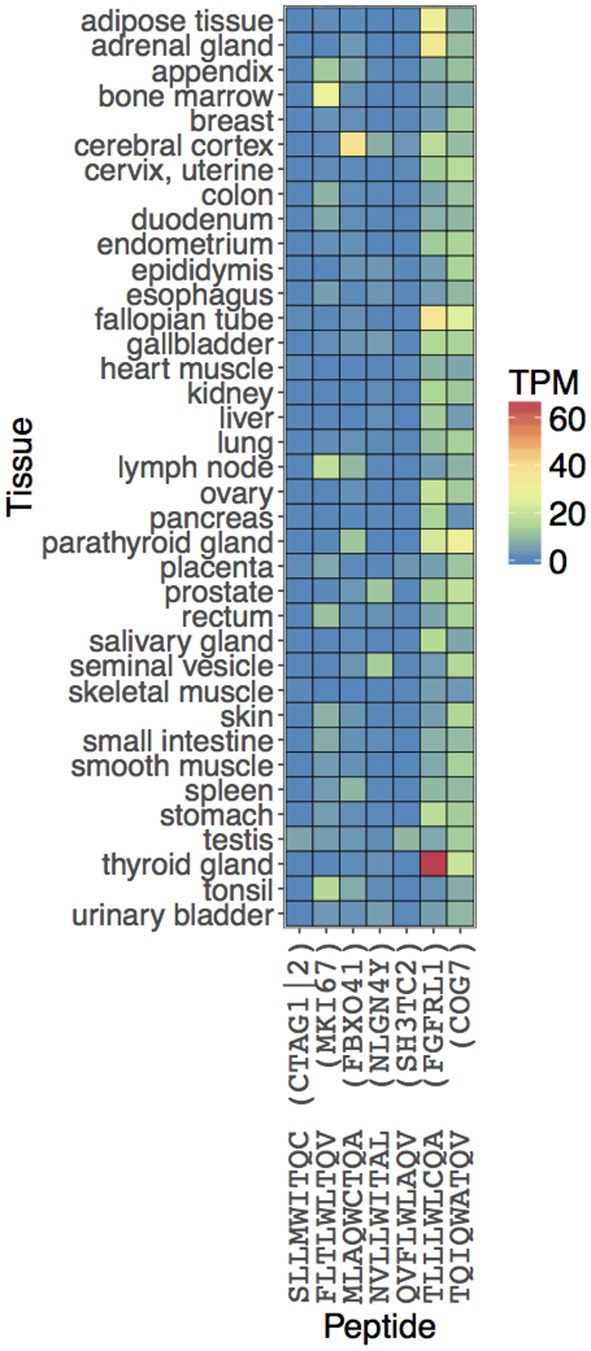
Estimating biological relevance of NY-ESO^c259^ TCR activating peptides. The expression values of the peptides are obtained from the associated genes. RNA levels in 37 tissues are depicted based on RNA-seq. Expression values are shown in Transcripts Per Million (TPM) units.

We computationally predicted and experimentally validated seven peptide sequences (including NY-ESO-1 antigen SLLMWITQC) capable of activating T cells through interaction with NY-ESO^c259^ TCR. These peptides, if naturally processed and presented on MHC molecules in normal human tissues, could potentially cause off-target effects in NY-ESO^c259^ TCR based adoptive cell therapies. To verify whether the predicted peptides are indeed presented and constitute a realistic off-target risk, we explored the publicly available gene expression and immunological data. First, we examined expression profiles of the genes serving as a source of peptides with high T cell activating potential in multiple normal tissues (Figure 5). Most of the genes demonstrated high RNA expression levels [Transcript Per Million (TPM) > 10] in at least several normal tissues, and the genes encoding two predicted peptides, TQIQWATQV (COG7) and TLLLWLCQA (FGFRL1), were highly expressed across a wide range of tissues. Secondly, we queried the peptide sequences against seven MHC ligands and T cell epitope databases (30, 41–46). We were not able to find the predicted off-target peptides, in spite of their high apparent RNA expression, potentially either reflecting that the peptides are not endogenously processed and presented on HLA^*^A02:01 molecules or due to limitations in assay sensitivity. This indicates that there is currently still a gap between the prediction of peptide ligands for a given MHC, and the actual detection and confirmation of the existence of such predicted peptides. As the sensitivity of the mass spectrometry HLA ligandome platform increases, integration of *in silico* predictive algorithms with high-quality HLA ligandome data will become a more and more accurate tool for identifying biologically relevant peptides with high TCR off-target potential.

## Discussion

Engineered T cells for adoptive therapies provide tumor-specific immunity to cancer patients (47). However, autologous T cells that have been genetically retargeted, particularly with engineered TCRs, have demonstrated a risk of on- and off-target toxicity. The main risk of genetically-modified, affinity-enhanced TCRs remains the recognition of peptide antigens presented on normal healthy tissues with sequence or structural similarity to the targeted peptide presented and recognized on target tumor cells (9, 13, 15). The importance of *in vitro* studies and the development of algorithms to predict potential off-target peptide recognition became apparent from the similarity of the peptides recognized by a TCR engineered to recognize Melanoma-associated Antigen 3 (MAGE-A3) (15). Further, lethal off-target recognition mediated by engineered TCRs has been reported in two patients with metastatic melanoma and multiple myeloma treated with an affinity-enhanced TCR generated for adoptive therapy against the HLA-A^*^01-restricted MAGE-A3 peptide EVDPIGHLY (13, 15). Further investigation demonstrated the unexpected cross-reactivity of the MAGE-A3 peptide with an HLA-A^*^01 restricted peptide derived from a contractile cardiac muscle titin peptide (ESDPIVAQY) presented in heart tissues (13). In a different trial, TCRs targeting the MAGE-A3/A9 HLA-A^*^02 epitope KVAELVHFL also recognized the MAGE-A12 epitope KMAELVHFL and, to a lesser extent, the MAGE-A2 (KMVELVHFL) and MAGE-A6 (KVAKLVHFL) epitopes. The unexpected expression of MAGE-A12 in the brain eventually resulted in neuronal toxicities in two patients treated with MAGE-A3 TCR-engineered T cells (14).

Preclinical screening assessments of newly identified engineered high-affinity TCRs are therefore needed in order to predict risks in subsequent clinical trials. Here, we present a safety assessment package based on *in vitro* cellular assays and computational biology algorithms to quickly assess the risk of off-target toxicity of selected TCRs by identifying the TCR recognition kernel—a unique pattern for each TCR showing its recognition of peptide-MHC complexes. We not only estimate which positions within the epitope sequence are important for TCR recognition, but also show how individual amino acid changes at each position influences TCR recognition. Moreover, each amino acid substitution is weighted based on multiple experimental measurements; therefore, we can estimate the contribution of individual amino acid changes to the TCR recognition on a continuous scale. While our method does not interrogate multiple simultaneous substitutions within the epitope sequence, which would involve generation of a peptide library of 9^20^ peptides in total, we do show that the measured first order changes can be exploited to find recognized peptides with up to seven amino acid changes.

We used three separate and distinct *in vitro* assays to assess peptide recognition: (1) the binding of the TCR NY-ESO^c259^ to the target peptide, (2) the activation of TCR-expressing T cells after interaction with peptide-MHC complex, and (3) the killing of the peptide presenting cells by TCR-expressing T cells. These three assays yielded similar but not identical results. Using a combined dataset, we identified that positions P5 and P8 of the epitope peptide sequence are crucial for TCR NY-ESO^c259^ recognition. The P5-centered peg in the middle of the peptide contacts TCR alpha CDR3, beta CDR3 and alpha CDR1 loops (22), correspondingly substitution of tryptophan (W) at P5 with any other amino acid results in a significant decrease of binding, activation and killing. Similarly, the TCR-facing glutamine (Q) is critical for binding, activation and killing. Other positions within the epitope peptide sequence tolerate more individual amino acid changes, indicating that they are less critical for NY-ESO^c259^ recognition. In general, we observed a high similarity across all three *in vitro* assays (binding, activation, and killing). In particular, the normalized experimental measurements from activation and killing assays demonstrated a strong linear relationship, indicating a dependence between T cell activation level and killing efficiency. Comparing binding with either activation or killing assays demonstrates a less linear dependence, and in many cases a binding level of at least 75% is required to trigger T cell activation and killing mechanisms. The associations between binding and activations (Figure 1C, top) and activation and killing (Figure 1C, bottom) are strong; nevertheless, there are outlier peptides whose investigation may reveal additional structural characteristics for an effective T cell response.

We developed a novel computational algorithm that uses *in vitro* generated experimental values from the three cellular assays (binding, activation, and killing) to construct Position Weight Matrices (PWMs) by assigning normalized experimental measurements from a set of 134 altered peptides (including epitope peptide) to each of the 20 amino acids at each position. The obtained PWMs were used for scoring NY-ESO^c259^ TCR recognition of the 336,921 potential human HLA^*^A02:01 binding 9-mer peptides. To validate the results, we selected and tested 85 peptides with low, intermediate and high predicted TCR recognition scores in a T cell activation assay using AKD10R3 as well as primary T cells. We found that 7 (including NY-ESO-1 antigen SLLMWITQC) out of 16 peptides with high predicted score demonstrated strong activation of primary T cells, whereas none of the peptides with low or intermediate scores showed evidence of T cells and AKD10R3 cell activation. Among the peptides with high TCR recognition score, activating peptides on average had a higher score (0.923) than non-activating (0.896) peptides and the difference was statistically significant (Wilcoxon two-sided test, *p* = 0.003). Based on this finding, we built a linear classifier discriminating between activating and non-activating peptides based on TCR recognition score. At a cutoff score of 0.905 the classifier yielded very high values of both sensitivity (1) and specificity (0.950). Overall, we found only 12 peptides (including NY-ESO-1 antigen SLLMWITQC) binding to HLA-A^*^02:01 with the TCR recognition score greater than the estimated cutoff, and 7 (including NY-ESO-1 antigen SLLMWITQC) of them showed strong activation of T cells. Notably, the activating peptides demonstrated unexpectedly high edit distance—the number of substitutions needed to transform query sequence to the epitope sequence, with most of the peptides being at least five edit operations away from the epitope sequence and with one peptide being seven edit operations away. This finding suggests that our computational algorithm based on experimental data from three *in vitro* assays can correctly identify potential off-target peptides, even those with a high degree of sequence dissimilarity to the original epitope sequence, thereby greatly reducing the risk for false-negative and missed epitopes.

The method described here enables identification of potential antigenic peptides with high TCR off-target recognition potential in a timely and reliable manner and allows for the identification of a focused set of peptides for downstream specificity testing. While we were able to use the algorithm to predict recognition of the TTN off-target epitope using alanine scan data, the PWM defined by alanine scan data has limited utility. The same method also enables “de-orphanization,” including identification of the off-target, HLA-binding, human peptides recognized by a given TCR. Further incorporation of RNA expression and high sensitivity MHC-I ligandome profiles from normal tissues will progressively allow for the reliable and precise identification of biologically relevant off-target antigens that are, or are not, expressed, processed and presented in disease-relevant and healthy tissues. Further delineation of the complete human healthy tissue and cancer MHC ligandome is likely to lead to a decrease in the need for extensive downstream validation *in vitro*. Excitingly, these results demonstrate that replacement library screening can be successfully used to assess recognition specificity of any TCR and enables selection of those TCRs with highly specific recognition kernels that are non-reactive to healthy human tissues. This will bring down the cost and time required for bringing such TCRs into clinical development.

## Data Availability Statement

Datasets generated for this study are included in the article/Supplementary Files.

## Author Contributions

AK, CC, VS, RS, JC, and MD participated in the design of the study. JC created the algorithm. AK and JC performed the computational data analysis. RS, VS, CC, JC, AK, and MD drafted the final manuscript. KW conducted and analyzed the cell-based assays. SW performed and analyzed the primary human cell-based assays. JJ generated and purified the soluble TCR. BJ synthesized peptides. DU analyzed structure data. All authors critically evaluated the manuscript.

### Conflict of Interest

The authors declare that the funding for this study was provided by the authors' employer, Agenus Inc. Agenus employees designed the study, analyzed the results, decided to publish the results, and wrote and reviewed the manuscript.

## Supplementary Material

Supplementary Table 1 An overview of the peptides selected for validation in AKD10R3 cells. The edit (Hamming) distance defines the minimum number of operations required to transform a peptide sequence into an epitope peptide sequence (SLLMWITQC). HLA-binding affinity (IC50, nM) was predicted by the netMHCpan v3.0 software package. The TCR recognition score was calculated by taking the mean of the predicted scores estimated for binding, activation and killing assays. AKD10R3 activation was measured by quantifying the percentage of EGFP positive cells (as described in Material and Methods). We subtracted background noise from experimental measurements and normalized individual values to the epitope peptide signal (SLLMWITQC). Negative values correspond to measurements below background noise level.
